# Prognostic determinants and mortality risk of advanced schistosomiasis revealed by Lasso-Cox regression integrative approach

**DOI:** 10.1371/journal.pntd.0013846

**Published:** 2026-01-05

**Authors:** Zhong Hong, Yinlong Li, Shiqing Zhang, Xiaojuan Xu, Ting Liu, Qilu Chen, Jing Xu

**Affiliations:** 1 School of Finance, Taxation and Public Administration, Tongling University, Tongling, China; 2 National Institute of Parasitic Diseases, Chinese Center for Disease Control and Prevention, Shanghai, China; 3 Chinese Center for Tropical Diseases Research, Shanghai, China; 4 National Key Laboratory of Intelligent Tracking and Forecasting for Infectious Diseases, NHC Key Laboratory of Parasite and Vector Biology, Shanghai, China; 5 WHO Collaborating Centre for Tropical Diseases, Shanghai, China; 6 National Center for International Research on Tropical Diseases, Ministry of Science and Technology, Shanghai, China; 7 Anhui Provincial Center for Disease Control and Prevention, Hefei, China; 8 School of Global Health, Chinese Center for Tropical Diseases Research, Shanghai Jiao Tong University School of Medicine, Shanghai, China; Universidad Nacional Autónoma de México, MEXICO

## Abstract

**Objective:**

To identify survival-related risk factors in patients with advanced schistosomiasis, develop a predictive model using integrative approach of LASSO-Cox regression, and construct a nomogram for visualizing the model’s risk prediction framework.

**Methods:**

Data from 628 advanced schistosomiasis patients treated at Dongzhi Schistosomiasis Hospital between 2019 and 2022 were retrospectively analyzed. LASSO regression was used to select variables associated with survival outcomes, which were subsequently incorporated into a Cox proportional hazards (CPH) model. Internal validation included assessments of discriminative ability (C-index, area under the receiver operating characteristic curve [AUC]), calibration (calibration curves), and clinical utility (decision curve analysis) to evaluate model performance. The final model was visualized via a nomogram depicting the risk prediction algorithm.

**Results:**

LASSO regression identified four independent predictors: carbohydrate antigen 125, hyaluronic acid, ascites grade Ⅱ, and ascites grade Ⅲ. The LASSO-Cox model exhibited strong discriminative performance, with a C-index of 0.886 (SE = 0.025) in the training set and 0.922 (SE = 0.025) in the validation set. Calibration curves showed excellent agreement between predicted and observed survival probabilities, and decision curve analysis confirmed clinical utility across a range of threshold probabilities. A nomogram was developed to translate the model into a user-friendly visual tool for risk stratification.

**Conclusions:**

The constructed nomogram serves as a practical tool for identifying advanced schistosomiasis patients at high mortality risk. Clinicians can leverage this model to tailor individualized follow-up and treatment strategies, potentially improving long-term outcomes by targeting interventions to patients with the greatest need.

## Author summary

Human schistosomiasis is a neglected tropical disease caused by trematode parasites of the genus Schistosoma. It is particularly dangerous for people who live in regions featured by inadequate access to sanitation and safe water, and predicting its severity is critical for timely intervention. This study aimed to develop a simple, user-friendly tool to survival-related risk factors in patients with advanced schistosomiasis. Through a retrospective analysis of clinical data from 628 patients with advanced schistosomiasis, we identified four critical factors- carbohydrate antigen 125, hyaluronic acid, ascites grade Ⅱ, and ascites grade Ⅲ, that can predict disease severity. Based on these four factors, we developed a nomogram tool to assist clinicians in quickly assessing the risk of patients with advanced schistosomiasis. The validation of the nomogram demonstrated its accuracy and utility in clinical decision-making. Our study suggests that this nomogram could serve as a valuable tool in healthcare, aiding physicians in identifying patients with advanced schistosomiasis of high mortality risk accurately, making more informed treatment decisions, and provide guidance for health administrative department to make management decisions to decrease the disease burden.

## Introduction

Human schistosomiasis is a neglected tropical disease caused by trematode parasites of the genus *Schistosoma* [1]. The disease persists as a public health threat in 78 countries and regions across Asia, South America, the Middle East, and Africa. Globally, over 780 million individuals are at risk of infection and 250 million people get infection of *Schistosoma* spp., while 90% of cases concentrated in sub-Saharan Africa regions featured by inadequate access to sanitation and safe water [2–4]. It accounts for approximately 280,000–500,000 annual deaths worldwide [5] and imposes a global burden of 3.31 million disability-adjusted life years (DALYs) [6], which highlights the significant consumption of global public health resources by the disease [7]. In China, schistosomiasis is caused by *S. japonicum*, which typically results in more severe pathological lesions due to the higher egg production of its adult worms [8], and is recognized as the most severe form of schistosomiasis [9]*.* Advanced schistosomiasis, the late stage of the disease, results in splenomegaly, ascites, portal hypertension, liver fibrosis, cirrhosis, upper gastrointestinal bleeding, hepatic failure, spontaneous bacterial peritonitis, disability, or even death [10,11].

Advanced schistosomiasis not only affects patients’ physical health, but also has a profound impact on their social and economic status, especially in poor areas with limited medical resources, where the disease often becomes one of the main causes of poverty and re-poverty [12]. As a longstanding endemic area for *Schistosoma japonicum,* China has been severely impacted by schistosomiasis japonica and bore the world’s heaviest disease burden of schistosomiasis japonica in the past [13]. National survey conducted in 1950’s estimated that 11.6 million people in China were infected with schistosomiasis [14]. After sustained control efforts implemented by the central government over seven decades, schistosome infections have decreased significantly [15]. China has shifted from transmission control to transmission interruption and elimination, with advanced schistosomiasis currently leading to the primary disease burden. The incidence of advanced schistosomiasis has been steadily increasing due to the persistent progress of the disease and lack of efficient therapeutic approaches [16]. In 2023, there were 27,768 patients of advanced schistosomiasis in China, and 1,942 advanced patients died from this disease in that year [17]. This poses a significant challenge to the elimination of schistosomiasis and the construction of a Healthy China by 2030, suggesting that the disease burden remains non-negligible [18]. In summary, the disease burden of schistosomiasis is very high, causing significant impact on individuals’ health, social and economic status, and public health, and is an urgent public health issue that needs to be addressed [19]. Therefore, it is essential to conduct in-depth research to identify prognostic factors for clinical outcomes.

Since early intervention is associated with improved prognosis [20], identifying patients at high risk of death will benefit the precision medicine to ensure that these patients receive appropriate treatment and long-term follow-up. Existing nomograms for advanced schistosomiasis [9,21] focus on either binary short-term outcomes or use traditional variable selection methods that may overlook clinically meaningful factors. A nomogram that integrates survival time data and robust variable screening to predict long-term mortality risk-with direct utility for clinical prognosis and policy-making-remains needed to address unmet gaps in current research.

To address this critical knowledge gap, our team previously developed a logistic regression model with 4 predictors (using a clinical dataset largely consistent with that of the present study) [21]. However, this prior study focused solely on predicting short-term mortality risk via logistic regression. Because it did not incorporate time-dependent variables, its ability to clarify the complex associations between prognostic outcomes and influencing factors was limited. The present study systematically extends this earlier work by specifically addressing unresolved questions in long-term prognostic assessment. As are well established, accurate prognostic evaluation forms the foundation of prevention and treatment; wherein clinical prognostic factors must be clearly identified [9]. In clinical research, survival analyses-such as the traditional CPH model-have been used to identify prognostic factors for clinical outcomes. However, these methods may be overly simplistic for complex clinical endpoints like progression to death. Additionally, traditional survival analysis models are prone to overfitting, compromising model accuracy due to potential multicollinearity among predictors. To address these limitations, this study introduced regularization techniques and spline regression, constructing a patient-based survival prediction model with internal validation. This approach aimed to provide important references for analyzing the epidemiological characteristics and patterns of advanced schistosomiasis, identifying patients with high mortality risk in a timely manner, formulating effective control strategies, guiding clinical follow-up protocols, and facilitating accurate prognostic assessment.

## Materials and methods

### Ethics statement

All patients participating in the study were required to provide written informed consent. And, this study was conducted according to the guidelines of the Declaration of Helsinki and approved by the Ethics Committee of National Institute of Parasitic Diseases of China (No.2022004). Furthermore, we ensured that all patient data were anonymized to maintain confidentiality and privacy, in accordance with ethical standards.

### Data source and study population

Dongzhi County (**S1 Fig**), situated in southern Anhui Province along the Yangtze River, is an endemic region for schistosomiasis japonica, characterized by both hill and marshland ecotypes. At the time of study enrollment, there were 800 patients with advanced schistosomiasis japonica currently receiving care at Dongzhi County, among which approximately 35 patients succumbing to the disease annually. According to the Diagnostic Criteria for Schistosomiasis (**WS261-2006**) promulgated by China’s Ministry of Health, patients are classified as advanced schistosomiasis when they met the following four criteria: (1) residence in or travel to endemic schistosomiasis japonica areas with confirmed contact with water infested by *Schistosoma japonicum*; (2) clinical manifestations including ascites, splenomegaly, portal hypertension, gastroesophageal variceal bleeding, granulomatous lesions of the colon/rectum, or severe growth retardation; (3) positive serological testing for *S. japonicum* antibodies (or serological negative but confirmed infection in history); and (4) pathological confirmation of infection via stool examination or rectal biopsy.

Demographic, clinical, laboratory, and ultrasound data of patients admitted to Dongzhi Schistosomiasis Hospital were collected between January 2019 and July 2022. Patient identifying information was strictly maintained confidential. All patients had the right to withdraw from the study without compromising their clinical rights or benefits.

### Inclusion and exclusion criteria of participants

The inclusion criteria for patients were as follows: (1) Provision of written informed consent for study participation; (2) Completeness of baseline demographic and clinical data; (3) A verified diagnosis of advanced schistosomiasis japonica; (4) Fulfillment of eligibility criteria for China’s Treatment and Assistance Program for Advanced Schistosomiasis Japonica.

The exclusion criteria were as follows: (1) Refusal of informed consent; (2) Incomplete baseline data, including demographic, clinical, laboratory, ultrasound, or survival outcome information; (3) Coexistence with overlapping clinical manifestations, such as primary hepatocellular carcinoma, primary hypersplenism, primary ascites, or primary liver fibrosis; (4) Ineligibility for China’s Treatment and Assistance Program for Advanced Schistosomiasis Japonica.

### Candidate variables for selection and prediction

A total of 31 variables were included: (1)Primary outcomes: vital status (death or survival), survival time; (2) Demographics: age, gender, occupation; (3) Clinical parameters: splenectomy, cholecystectomy, hypertension, hypoalbuminemia, hypokalemia, gastrointestinal bleeding, coagulopathy, diabetes, hepatic encephalopathy, anemia grade, body mass index; (4) Laboratory parameters: HBV infection status, albumin, total protein, high-density lipoprotein, carbohydrate antigen 125, hyaluronate, laminin, N-terminal procollagen III peptide, type IV collagen, total bilirubin, direct bilirubin. (5) Ultrasonic findings: liver fibrosis stage, right liver atrophy, gallbladder pathology, ascites grade. Patients were followed up until the study’s end date. The primary endpoint was all-cause mortality either during hospitalization or after discharge.

**Fig 1:**
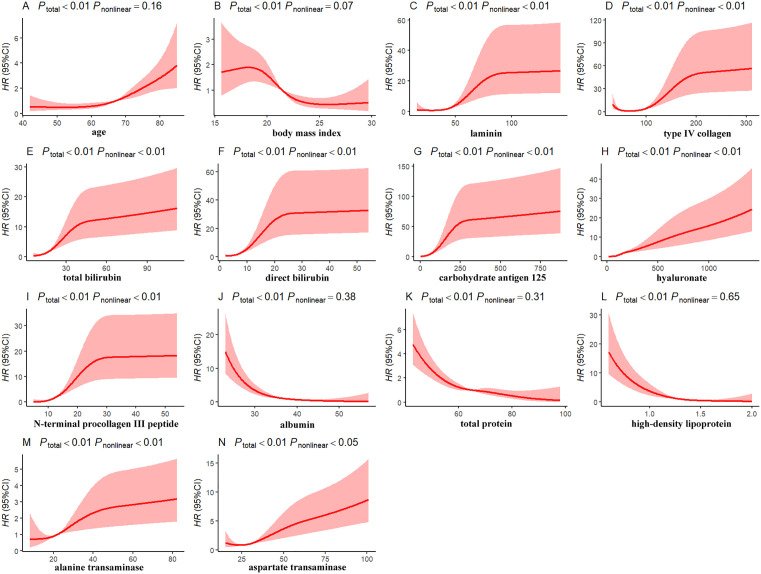
Potential threshold associations between variables.

Restricted cubic splines (RCS) with 4 knots placed at the 5th, 25th, 75th, and 95th percentiles of each variable were used to evaluate potential nonlinear associations between continuous covariates (age, total bilirubin, direct bilirubin, carbohydrate antigen 125, hyaluronate, laminin, N-terminal procollagen III peptide, body mass index, type IV collagen, albumin, total protein, high-density lipoprotein, alanine transaminase, aspartate transaminase) and mortality risk. Ten indices (age, total bilirubin, direct bilirubin, carbohydrate antigen 125, hyaluronic acid, laminin, N-terminal procollagen III peptide, type IV collagen, alanine transaminase, aspartate transaminase) demonstrated significant threshold effects (nonlinearity *P* < 0.05) and were classified into distinct groups based on RCS-derived inflection points (**Fig 1**). The remaining four indices (body mass index, albumin, total protein, high-density lipoprotein) were categorized as normal or abnormal according to established medical reference ranges. In this study, all multicategorical variables were converted into dummy variables based on medical reference ranges or RCS-derived inflection points, and a total of 42 variables were ultimately incorporated into the model development.

### Establishment of training set and validation set

Patients were randomly assigned to training and validation datasets in a 7:3 ratio, ensuring balanced e distribution of outcome events and covariates between the two groups. The training dataset was used to screen predictors and develop the model, while the internal validation dataset was employed to assess model performance.

### Model derivation

**Fig 2:**
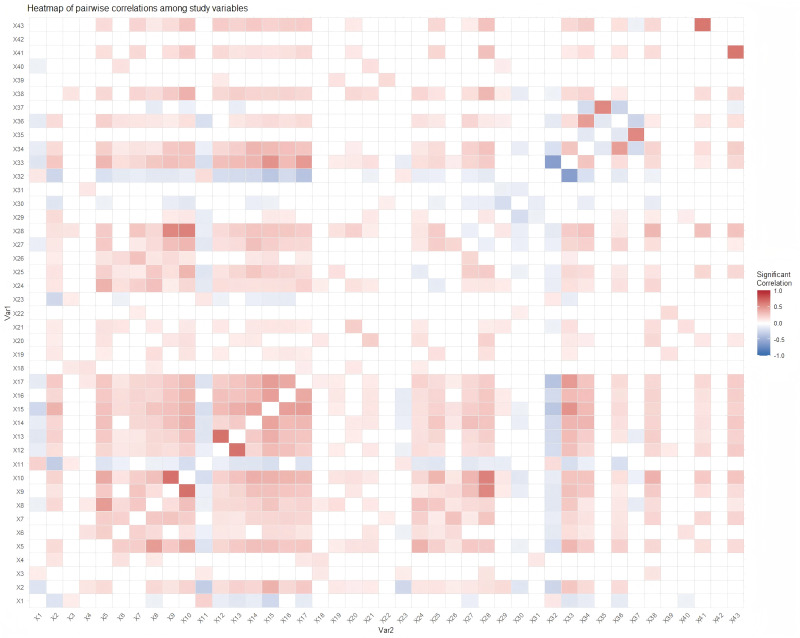
Heatmap of pairwise correlations among study variables. *Pairwise correlation coefficients are displayed within the figure, with positive/negative values denoting positive/negative associations.

Statistical analyses were conducted using SPSS version 25.0 (SPSS Inc., Chicago, IL, USA) and R software version 5.0 (https://www.r-project.org, accessed 2 July 2022). Descriptive statistics were used to characterize baseline characteristics in both the model derivation and internal validation datasets. Categorical variables were compared using the chi-squared test. All *p*-values were two-tailed, with statistical significance defined as *p* < 0.05. First, pairwise correlation coefficients among variables were calculated and visualized via a heatmap (**Fig 2**). The heatmap indicated strong inter-variable correlations, which could potentially introduce model complexity, interpretability challenges, and diminished performance. To address this, penalized regression was applied for variable selection.

The penalized regression approach was adopted in accordance with the Transparent Reporting of a Multivariable Prediction Model for Individual Prognosis or Diagnosis (TRIPOD) checklist for developing and validating risk prediction models [22]. Regularization involves incorporating a penalty term into the objective function, which serves to control model complexity by shrinking the magnitude of regression coefficients. When the shrinkage is enforced to exactly zero via this penalty, it is referred to as the L1 norm or L1 penalty [23,24]. The least absolute shrinkage and selection operator (LASSO) employs an L1 penalty, with the penalty strength governed by a regularization parameter (λ). This parameter was optimally selected using a tenfold cross-validation procedure [25], which minimizes bias from specific data partitioning schemes. A Cox proportional hazards (CPH) model was constructed using candidate predictors filtered by LASSO regression. Final predictor selection was performed via backward elimination based on the Akaike information criterion (AIC). Additionally, variance inflation factors (VIFs) were calculated to assess multicollinearity among variables, with VIF values > 4.0 indicating significant collinearity. Variables exceeding this threshold were excluded from the final model to ensure interpretability and statistical validity.

### Assessment of model performance

The performance of the established model was evaluated in terms of discriminative ability, calibration performance and clinical utility [26]. Briefly, discriminative ability was assessed via the concordance index (C-index, equivalent to the area under the receiver operating characteristic (ROC) curve in binary logistic regression), calculated through bootstrapping with 1000 resamples [27]. The C-index ranges from 0.5(random prediction) to 1.0 (perfect discrimination), with values > 0.7 indicating reasonable predictive performance. Calibration performance was evaluated using calibration plots, which visualize the agreement between observed and predicted outcomes. A calibration curve approximating the ideal 45° line indicated close correspondence between model predictions and actual observations. Clinical utility was assessed via decision curve analysis (DCA), a methodology designed to quantify the net clinical benefit of nomograms across a spectrum of threshold probabilities. Two reference curves were plotted: the “treat-all-patients” strategy (representing maximal clinical intervention and associated costs) and the “treat-none” strategy (denoting no intervention and absence of clinical benefit) [28].

## Results

### Demographic information of participants

Of the 860 patients enrolled in the database, 628 met the inclusion criteria for final analysis (**S1 Table**). These were randomly assigned to the training set (n = 440) and internal validation set (n = 180), No significant differences in baseline variables were observed between two sets (all *p* > 0.05; **Table 1**). The training set comprised 195 males and 245 females, with 184 individuals younger than 67 years and 256 aged 67 years or older. The validation set included 84 males and 104 females, of whom 75 were younger than 67 years and 113 were 67 years or older.

**Table 1:** Characteristics of patients with advanced schistosomiasis in this study.

Variables	Assigned variables	Categories	Training Set	Validation Set	*P*-Value
Survival	Y	0 = live	379 (86.1%)	167 (88.8%)	0.61
		1 = death	61 (13.9%)	21 (11.2%)	
Gender^#^	X1	0 = male	195 (44.3%)	84 (44.7%)	1.00*
		1 = female	245 (55.7%)	104 (55.3%)	
Age^#^	X2	0 = “<67”	184 (41.8%)	75 (39.9%)	0.72
		1 = “≥67”	256 (58.2%)	113 (60.1%)	
Cholecystectomy^#^	X3	0 = no	374 (85%)	155 (82.4%)	0.49
		1 = yes	66 (15%)	33 (17.6%)	
Hypertension^#^	X4	0 = no	309 (70.2%)	129 (68.6%)	0.76
		1 = yes	131 (29.8%)	59 (31.4%)	
Hypoalbuminemia^#^	X5	0 = no	326 (74.1%)	144 (76.6%)	0.57
		1 = yes	114 (25.9%)	44 (23.4%)	
Hypokalemia^#^	X6	0 = no	377 (85.7%)	168 (89.4%)	0.26
		1 = yes	63 (14.3%)	20 (10.6%)	
Gastrointestinal bleeding^#^	X7	0 = no	414 (94.1%)	179 (95.2%)	0.71
		1 = yes	26 (5.9%)	9 (4.8%)	
Coagulopathy^#^	X8	0 = no	326 (74.1%)	138 (73.4%)	0.94
		1 = yes	114 (25.9%)	50 (26.6%)	
Liver fibrosis^#^	X9	0 = no	379 (86.1%)	165 (87.8%)	0.67
		1 = yes	61 (13.9%)	23 (12.2%)	
Atrophy of the right liver ^#^	X10	0 = no	371 (84.3%)	162 (86.2%)	0.64
		1 = yes	69 (15.7%)	26 (13.8%)	
Splenectomy^#^	X11	0 = no	194 (44.2%)	76 (40.4%)	0.43
		1 = yes	245 (55.8%)	112 (59.6%)	
Total bilirubin ^#^	X12	0 = “<14”	193 (43.9%)	85 (45.2%)	0.82
		1 = “≥14”	247 (56.1%)	103 (54.8%)	
Direct bilirubin ^#^	X13	0 = “<5”	188 (42.7%)	79 (42%)	0.94
		1 = “≥5”	252 (57.3%)	109 (58%)	
Carbohydrate antigen 125^#^	X14	0 = “<17”	216 (49.1%)	89 (47.3%)	0.75
		1 = “≥17”	224 (50.9%)	99 (52.7%)	
Hyaluronic acid ^#^	X15	0 = “<125”	228 (51.8%)	86 (45.7%)	0.19
		1 = “≥125”	212 (48.2%)	102 (54.3%)	
Laminin ^#^	X16	0 = “<39”	221 (50.2%)	90 (47.9%)	0.65
		1 = “≥39”	219 (49.8%)	98 (52.1%)	
Procollagen III N-terminal peptide ^#^	X17	0 = “<11”	220 (50%)	93 (49.5%)	0.97
		1 = “≥11”	1 = “≥39”	95 (50.5%)	
Diabetes^#^	X18	0 = no	409 (93%)	175 (93.1%)	1.00*
		1 = yes	31 (7%)	13 (6.9%)	
Hepatitis B virus infection status ^#^	X19	0 = no	430 (97.7%)	181 (96.3%)	0.45
		1 = yes	10 (2.3%)	7 (3.7%)	
Hepatic encephalopathy^#^	X20	0 = no	435 (98.9%)	187 (99.5%)	0.67
		1 = yes	5 (1.1%)	1 (0.5%)	
Other cancer^#^	X21	0 = no	431 (98%)	185 (98.4%)	1.00*
		1 = yes	9 (2%)	3 (1.6%)	
Occupation^#^	Ref	0 = farmer	416 (94.5%)	169 (89.9%)	0.06
	X22	1 = fisher	1 (0.2%)	2 (1.1%)	
	X23	1 = other	23 (5.2%)	17 (9%)	
Anemia grade^#^	Ref	0 = normal	321 (73%)	150 (79.8%)	0.11
	X24	1 = Ⅰ grade	80 (18.2%)	20 (10.6%)	
	X25	1 = Ⅱ grade	31 (7%)	15 (8%)	
	X26	1 = Ⅲ grade	8 (1.8%)	3 (1.6%)	
Ascites grade^#^	Ref	0 = Ⅰ grade	359 (81.6%)	154 (81.9%)	0.98
	X27	1 = Ⅱ grade	46 (10.5%)	20 (10.6%)	
	X28	1 = Ⅲ grade	35 (8%)	14 (7.4%)	
Body mass index ^#^	Ref	0 = “18.5 ≤BMI < 23.9”	273 (62%)	102 (54.3%)	0.14
	X29	1 = “<18.5”	68 (15.5%)	28 (14.9%)	
	X30	1 = “23.9 <=BMI < 27.9”	80 (18.2%)	49 (26.1%)	
	X31	1 = “≥27.9”	19 (4.3%)	9 (4.8%)	
Type IV collagen ^#^	Ref	0 = “58 ≤CIV < 76”	95 (21.6%)	51 (27.1%)	0.23
	X32	1 = “<58”	112 (25.5%)	50 (26.6%)	
	X33	1 = “≥76”	233 (53%)	87 (46.3%)	
Albumin ^#^	Ref	0 = “36 ≤ALB < 55”	238 (54.1%)	111 (59%)	0.23
	X34	1 = “<36”	188 (42.7%)	75 (39.9%)	
	X35	1 = “≥55”	14 (3.2%)	2 (1.1%)	
Total protein ^#^	Ref	0 = “65 ≤TP < 85”	207 (47%)	91 (48.4%)	0.82
	X36	1 = “<65”	200 (45.5%)	81 (43.1%)	
	X37	1 = “≥85”	33 (7.5%)	16 (8.5%)	
High-density lipoprotein #	Ref	0 = “0.9 ≤HDL < 2.0”	408 (92.7%)	169 (89.9%)	0.38
	X38	1 = “<0.9”	26 (5.9%)	14 (7.4%)	
	X39	1 = “≥2.0”	6 (1.4%)	5 (2.7%)	
Alanine aminotransferase ^#^	Ref	0 = “< 22”	401 (91.1%)	169 (89.9%)	0.62
	X40	1 = “≥ 22”	39 (8.9%)	19 (10.1%)	
Aspartate aminotransferase ^#^	Ref	0 = “18 ≤AST < 29”	409 (93%)	172 (91.5%)	0.65
	X41	1 = “<18”	1 (0.2%)	0 (0%)	
	X42	1 = “≥29”	30 (6.8%)	16 (8.5%)	

# Frequency and proportion; chi-square test was used to compare differences between groups. * The value was close to 1.

### Variables selection

**Fig 3:**
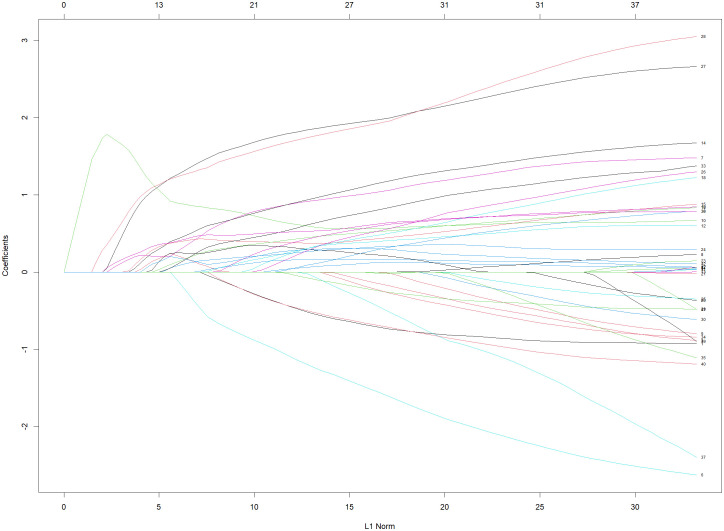
Plot for LASSO regression analysis. *The plot illustrates coefficients on the y-axis and the penalty term (λ) on the x-axis. Each colored line depicts the trajectory of a variable’s coefficient as the penalty term increases, with most coefficients being regularized to zero under stronger regularization.

**Fig 4:**
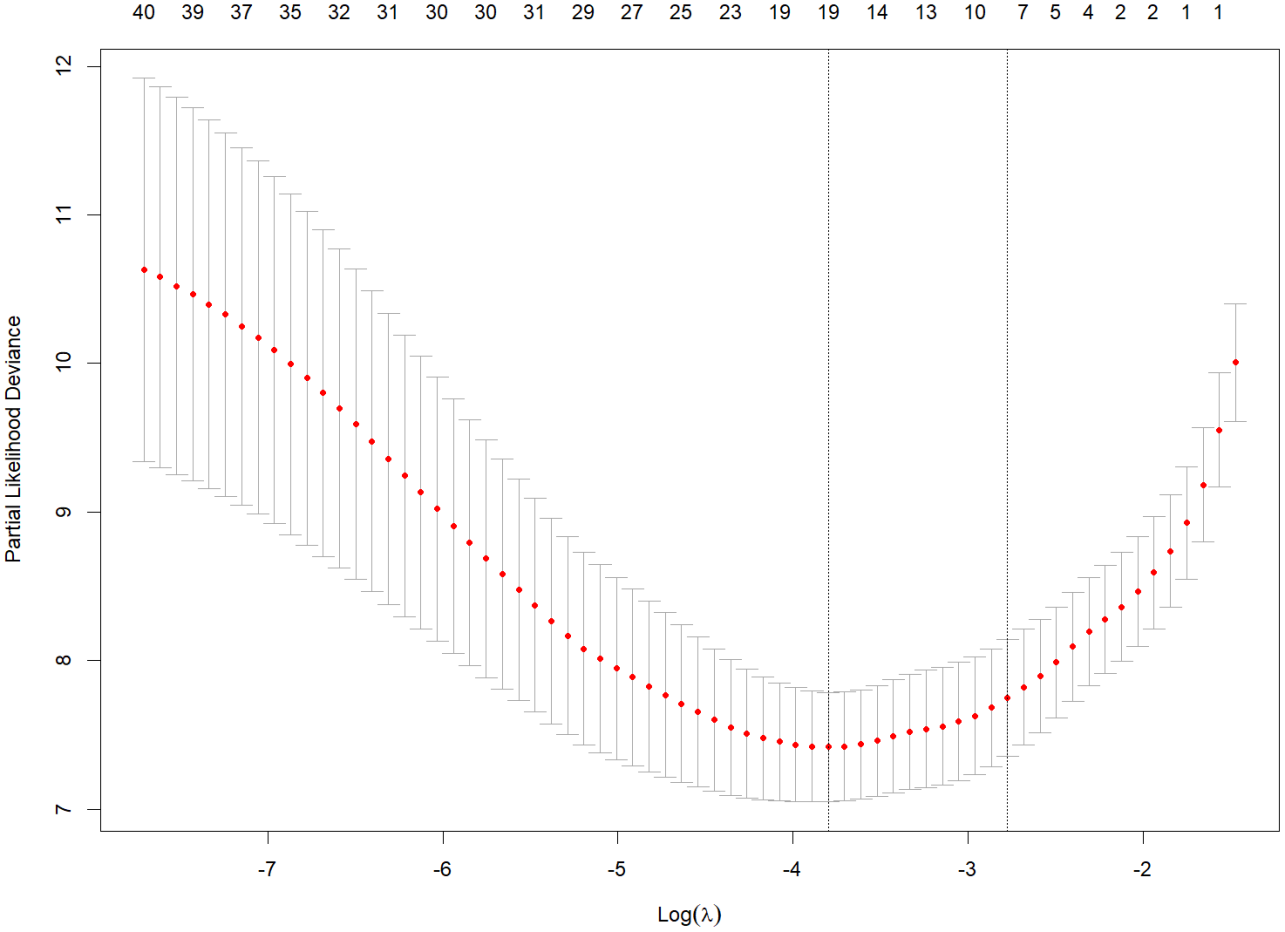
10-fold cross-validation process for selecting the penalty term (λ) by LASSO model. *The tuning parameter λ was chosen based on the minimum criteria, with dotted vertical lines denoting optimal values derived from two criteria: the minimum binomial deviance (minimum criteria) and the largest λ within one standard error (1-SE) of the minimum deviance (1-SE criteria). These criteria selected 19 variables and 8 variables, respectively.

Following the conversion of multi-categorical variables into dummy variables, 42 variables were incorporated into the LASSO regression analysis (**Fig 3**). The regularization parameter λ was selected via 10-fold cross-validation (**Fig 4**), yielding two candidate values: one corresponding to the minimum binomial deviance (19 variables; former line) and the other representing the largest λ within one standard error (SE) of the minimum deviance (8 variables; latter line). The latter λ [0.0568, log(λ) = −2.869] was chosen over the former [0.0004, log(λ) = −7.707)] because it imposed a stricter penalty, thereby reducing the number of variables more parsimoniously. Ultimately, eight variables-X7, X9, X10, X14, X15, X27, X28, and X38-were selected based on the regression results (**Table 2**).

**Table 2:** The coefficients screened by the LASSO regression.

Variable	Coefficient	Variable	Coefficient	Variable	Coefficient
X1	•	X15	0.16	X29	•
X2	•	X16	•	X30	•
X3	•	X17	•	X31	•
X4	•	X18	•	X32	•
X5	•	X19	•	X33	•
X6	•	X20	•	X34	•
X7	0.22	X21	•	X35	•
X8	•	X22	•	X36	•
X9	0.03	X23	•	X37	•
X10	1.35	X24	•	X38	0.26
X11	•	X25	•	X39	•
X12	•	X26	•	X40	•
X13	•	X27	0.89	X41	•
X14	0.12	X28	0.98	X42	•

The symbol “•” indicates coefficients shrunk to zero via regularization. Variable definitions: X7, gastrointestinal bleeding; X9, liver fibrosis; X10, atrophy of the right liver; X14, carbohydrate antigen 125; X15, hyaluronic acid; X27, ascites grade Ⅱ; X28, ascites grade Ⅲ; X38, high-density lipoprotein.

### Fitted model and constructed nomogram

**Fig 5:**
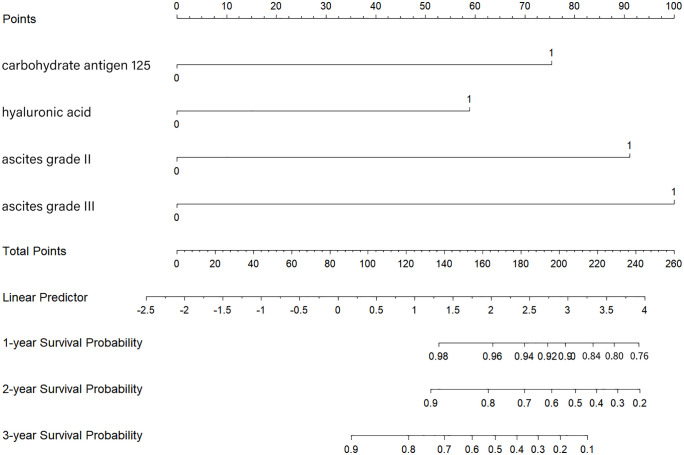
Established nomogram for predicting clinical outcomes in patients.

A Cox proportional hazards (CPH) model was constructed based on eight independent variables identified via LASSO regression. As shown in **Table 3**, four variables (X7, X9, X10, X38) were excluded from further analysis due to nonsignificant associations (*p* > 0.05). Subsequently, the model was fitted with the remaining four variables-carbohydrate antigen 125 (X14), hyaluronic acid (X15), ascites grade Ⅱ (X27), and ascites grade Ⅲ (X28)-as presented in **Table 4**. The final model incorporating these independent predictors was developed into a nomogram, which is visualized in **Fig 5**.

**Table 3:** Model based on eight independent variables.

Variable	exp (coefficient)	Exp (coefficient) 95% CI	*P*-Value
X7	1.42	0.75-2.69	0.28
X9	1.44	0.75-2.76	0.27
X10	1.39	0.63-3.10	0.41
X14	6.38	1.38-29.57	0.02*
X15	3.66	1.17-11.46	<0.001***
X27	6.17	2.47-15.43	<0.001***
X28	5.96	2.17-16.33	<0.001**
X38	1.61	0.85-3.06	0.14

Significance codes: *** 0.001; ** 0.01; * 0.05.

**Table 4:** Model based on four independent variables.

Variable	exp (coefficient)	Exp (coefficient) 95% CI	*P*-Value
X14	6.56	1.43-30.09	0.02*
X15	4.33	1.45-13.00	0.01**
X27	9.69	4.55-20.63	<0.001***
X28	12.09	5.78-25.30	<0.001***

Significance codes: *** 0.001; ** 0.01; * 0.05. X14, carbohydrate antigen 125; X15, hyaluronic acid; X27, ascites grade Ⅱ; X28, ascites grade Ⅲ.

### Assessment of nomogram

**Fig 6:**
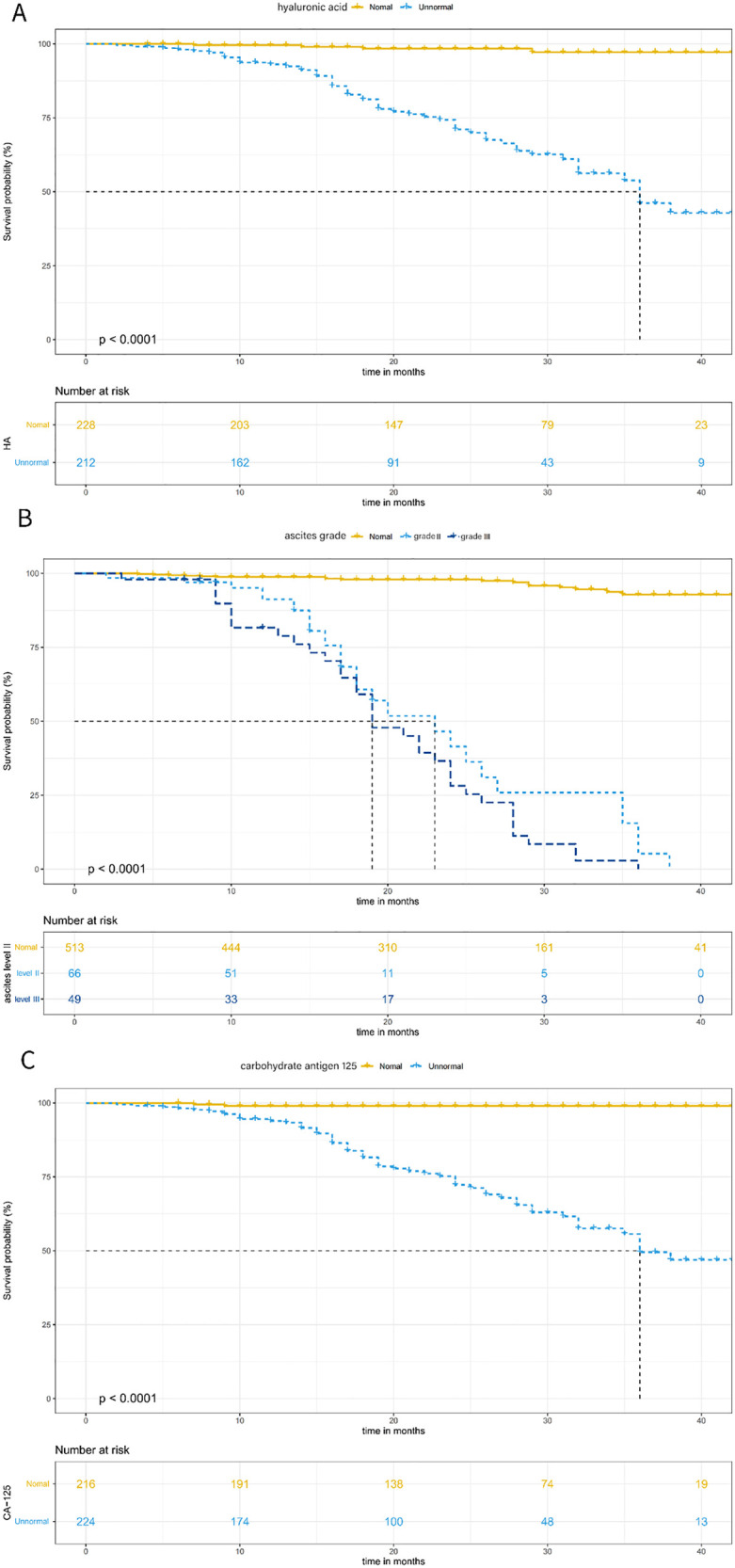
Kaplan–Meier survival curves stratified by hyaluronic acid grade, ascites grade, and carbohydrate antigen 125 grade in the training set, with log-rank test results for mortality differences. **(A)** Stratification by hyaluronic acid grade; **(B)** stratification by ascites grade; **(C)** stratification by carbohydrate antigen 125 grade.

**Fig 7:**
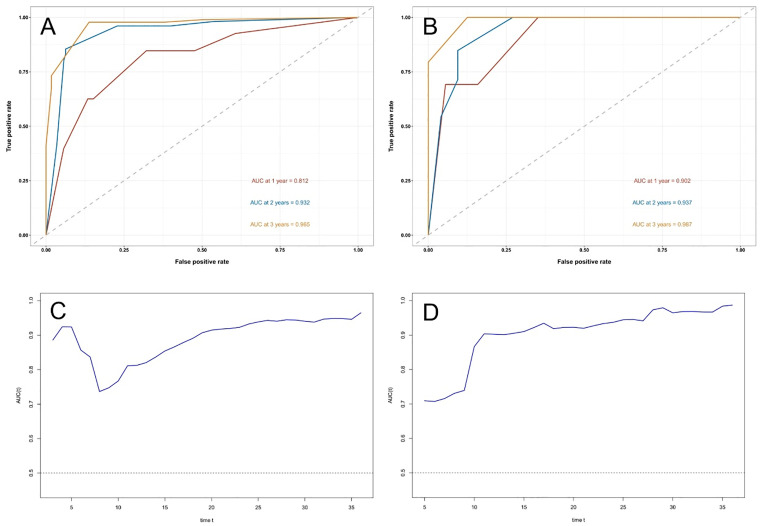
ROC curve of the nomogram. Receiver operating characteristic (ROC) curves **(A-B)** and time-dependent area under the curve (AUC) for 3-year overall survival (OS) prediction **(C-D)** using the nomogram. Time-dependent AUC values for predicting 3-year OS probability in the training and validation cohorts are shown in panels C and D, respectively.

**Fig 8:**
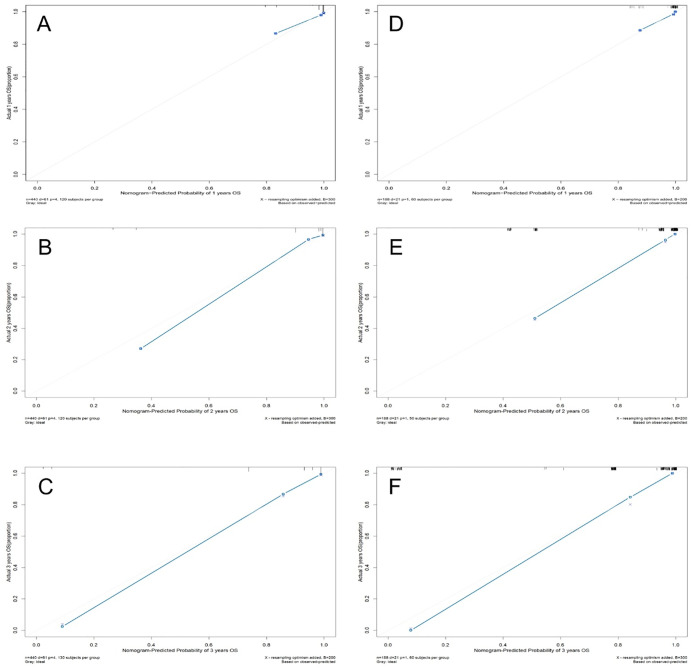
Calibration curves for 1-, 2-, and 3-year overall survival (OS) predictions. **(A–C)** Training set calibration curves for 1-year, 2-year, and 3-year OS; **(D–F)** validation set calibration curves for corresponding timepoints. The diagonal line represents the ideal reference where predicted survival probabilities align with observed outcomes. Blue dots, derived from bootstrap resampling (1000 resamples), illustrate the nomogram’s performance at each timepoint. The closer the light blue calibration curve approaches the diagonal line, the better the model’s agreement between predicted and observed survival probabilities.

The Kaplan–Meier curves showed that the overall survival rate reduced significantly in patients with abnormal hyaluronic acid, ascites grade, and carbohydrate antigen 125 (all *P* < 0.001, **Fig 6**). Moreover, as follow-up duration increased, the survival probability of patients with ascites grade III was lower than that of those with ascites grade II (**Fig 6**). The C-index was 0.886 (SE = 0.025) in the training set and 0.922 (SE = 0.025) in the validation set, showing strong discriminative ability of established model. The areas under the ROC curves both exceeded 0.8, and the time-dependent AUC for predicting 3-year overall survival (OS) was > 0.7 in both sets (**Fig 7**), indicating the nomogram’s favorable predicative performance. The calibration curves demonstrated high consistency between predicted and observed survival probabilities in both sets (**Fig 8**).

### Clinical value of the nomogram

**Fig 9:**
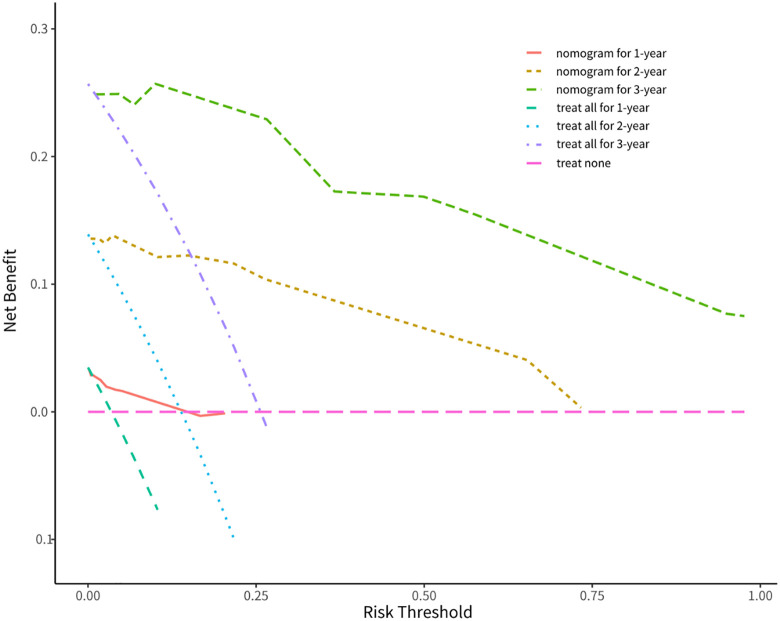
Clinical decision curves for 1-, 2-, and 3-year overall survival (OS) predictions. *The y-axis represents net clinical benefit. The orange, khaki, and green lines correspond to the nomogram’s 1-year, 2-year, and 3-year OS predictions, respectively. The pink line denotes the “treat-none” strategy (no intervention), while the gray line represents the “treat-all” strategy (universal intervention). Net benefit was calculated by subtracting the proportion of false-positive results from the proportion of true-positive results, weighted by the relative harm of forgoing treatment versus the negative consequences of unnecessary treatment [29]. Net benefit is a key metric in Decision Curve Analysis (DCA), calculated as ‘(True Positive Rate * Weight) - (False Positive Rate * (1-Weight))’; here, ‘Weight’ reflects the clinical emphasis on ‘avoiding missed diagnoses.’ The unit of net benefit in this study is ‘net benefit per 100 patients’-it is neither a percentage nor an improvement in survival probability. Instead, it represents the additional number of beneficial clinical decisions (e.g., correctly identifying patients who need intervention while avoiding unnecessary intervention for low-risk patients) achieved by our model compared to the ‘treat all’ or ‘treat none’ strategies at a specific threshold probability. Negative net benefit indicates that using the model to guide decisions at this threshold results in lower clinical utility than ‘not using any predictive model’ (i.e., the harm of unnecessary interventions exceeds the benefit of avoiding missed diagnoses.

Decision curve analysis (**Fig 9**) demonstrated that the nomogram’s predicted death probabilities yielded greater net clinical benefit than the “treat-none” or “treat-all” strategies across threshold probabilities. At a 10% decision threshold (i.e., treatment is initiated when the predicted death probability≥10%), the net benefit was 0.02 for 1-year outcomes, 0.13 for 2-year outcomes, and 0.25 for 3-year outcomes. Clinicians could apply the nomogram to determine whether treatment would provide greater benefit than non-intervention (“treat-none”) or universal intervention (“treat-all”) strategies at specific risk threshold.

## Discussion

The disease burden of advanced schistosomiasis primarily stems from its advanced stage, which is associated with liver fibrosis, cirrhosis, ascites, portal hypertension, splenomegaly, and gastroesophageal varices. These complications contribute to disability, loss of workforce participation and self-care ability, and even mortality [2,30,31]. Previous studies have demonstrated that advanced schistosomiasis japonica is linked to high morbidity, mortality, poor self-reported quality of life, and significant disability [11,32]. A report by Zhang et al. [17] noted that advanced schistosomiasis has been the predominant form in China for consecutive years, highlighting that effective management, follow-up, and intervention for previously infected individuals can delay or prevent pathological progression due to schistosomiasis. However, existing literature on survival analysis in patients with advanced schistosomiasis remains limited, leaving a gap in evidence to inform clinical practice [9].

Exploring prognostic factors and predicting patient outcomes can help clinicians identify individuals at high risk of adverse prognoses who require targeted attention and interventions. In this study, we employed a retrospective cross-sectional design and a LASSO-Cox proportional hazards (CPH) model to investigate the associations between prognostic outcomes in advanced schistosomiasis and population-based demographic, clinical, laboratory, and ultrasonic data. Prognostic factors-including carbohydrate antigen 125, hyaluronic acid, and ascites grades Ⅱ and Ⅲ, were selected to construct a nomogram for predicting mortality risk. Following performance evaluation, the developed model emerged as a practical tool for clinicians to identify advanced schistosomiasis patients at high risk of mortality, while also providing a theoretical basis for treatment planning and reducing the disease burden of schistosomiasis [33].

In our prior study, we found that ascites grade in patients with advanced schistosomiasis was significantly associated with mortality risk [21]. Ascites, defined as excessive fluid accumulation in the peritoneal cavity, represents the most common clinical manifestation of advanced hepatic disease. As a dominant complication of liver-specific damage, the severity of ascites directly influences overall prognosis. For example, the 1-year survival rate in cirrhotic patients with ascites is only 60%, while in those with refractory ascites, the 6-month survival rate drops below 50% [34]. Severe ascites also had been identified as one of the strongest predictors of heightened disability in advanced schistosomiasis [11]. Consistent with previous prognostic studies of advanced schistosomiasis [35], our analysis included severe ascites as a key prognostic factor in the nomogram model. The pathophysiology of ascites involves mechanisms such as portal hypertension, hypoalbuminemia, excessive fluid production, and lymphatic obstruction [2], with common etiologies including liver cirrhosis, malignancies, or heart failure [36]. As a chronic debilitating condition, ascites profoundly impairs patients’ quality of life [37]. Disease progression often leads to complications like bacterial peritonitis-manifesting as persistent fever and potentially severe infection-or acute heart failure. Additionally, massive ascites causes physical discomfort, including abdominal distension, pain, anorexia, and fatigue [38,39], while also restricting mobility [40] and altering body image, which can exacerbate psychological distress and further diminish quality of life [41]. Regrettably, timely diagnosis and access to effective treatment remain unavailable for all patients with advanced schistosomiasis.

In addition, this study identified carbohydrate antigen 125 and hyaluronic acid as independent predictors of mortality risk among various biochemical markers. As reported by Chen et al. [42], hyaluronic acid exhibits strong diagnostic utility for cirrhosis, outperforming liver fibrosis markers alone in distinguishing cirrhotic states. Furthermore, hyaluronic acid testing facilitates early detection of schistosomiasis-associated liver fibrosis. When combined with the four-item liver fibrosis panel, it improves diagnostic accuracy and sensitivity for liver fibrosis. This approach also differentiates between advanced and chronic schistosomiasis, providing critical guidance for precision treatment strategies [43]. Research has shown that carbohydrate antigen 125 grades correlate closely with prognosis in cirrhotic patients with ascites, where elevations signal disease severity and poor outcomes [44,45]. Additionally, rectosigmoid carcinoma in the context of schistosomiasis is associated with higher carbohydrate antigen 125 values even at early tumor stages [46], suggesting its potential role as a marker of microenvironmental changes in advanced schistosomiasis. We hypothesize that elevated carbohydrate antigen 125 may reflect decompensated liver function and increased oncogenic risk in these patients. Regular monitoring the dynamics of hyaluronic acid and carbohydrate antigen 125, in combination with early intervention upon biomarker abnormalities, could potentially delay progression to severe complications such as malignant ascites, cirrhosis, oncogenesis, and hepatic encephalopathy.

Therefore, intervention strategies based on nomogram predictions and the timing of interventions-these points are critical to ensuring the tool’s practical relevance. The nomogram developed in this study not only quantifies the survival risk of patients with advanced schistosomiasis japonica but also provides targeted references for formulating clinical intervention strategies. Based on risk stratification predicted by the nomogram, the following differentiated interventions are recommended: For high-risk patients (e.g., predicted 3-year survival rate < 30%), who often present with key risk factors such as persistent massive ascites, as well as significantly elevated levels of carbohydrate antigen 125 and hyaluronic acid, clinical management should focus on intensive strategies: ① Shorten the follow-up interval to once every quarter, combining abdominal imaging examinations (e.g., ultrasound or CT) and monitoring of hematological indices (carbohydrate antigen 125, hyaluronic acid, etc.) to early identify signs of disease decompensation; ② Implement targeted therapy for reversible risk factors, such as initiating antiviral treatment for patients with concurrent chronic hepatitis B to slow the progression of liver fibrosis; ③ Conduct prophylactic intervention for complications, such as standardized use of beta-blockers in patients with portal hypertension to reduce the risk of variceal bleeding; ④ Establish a multidisciplinary team consisting of hepatologists and infectious disease specialists to integrate expertise in parasitic disease management and end-stage liver disease treatment, optimizing comprehensive diagnosis and treatment plans. For low-risk patients (e.g., predicted 3-year survival rate > 80%), whose condition is generally in a relatively stable state, intervention focuses on maintaining disease stability and avoiding overtreatment: ① Adopt a standard follow-up frequency (once every year) to balance monitoring needs and medical resource consumption; ② Strengthen patient education to guide them in identifying warning symptoms such as jaundice, abdominal distension and ascites, improving self-reporting awareness; ③ Optimize basic care measures, including nutritional support (e.g., high-protein diet) and avoidance of hepatotoxic drugs (e.g., certain non-steroidal anti-inflammatory drugs), to reduce triggers for disease deterioration.

Furthermore, it should be clarified that this nomogram is not intended to advocate delaying basic treatment for patients with advanced schistosomiasis japonica. All diagnosed patients should receive standard baseline interventions (e.g., praziquantel for antiparasitic treatment, basic liver-protective therapy, etc.) promptly after diagnosis, which is the premise of disease management. The core value of the nomogram lies in optimizing the intensity and timing of interventions on top of baseline treatment. Although advanced schistosomiasis is a progressive disease, the rate of progression varies significantly among individuals: some patients can maintain mild symptoms for a long time, while others rapidly progress to decompensated cirrhosis within 1–2 years. Through risk stratification, the nomogram enables “precision intervention”: initiating intensive measures for high-risk patients in advance (e.g., starting ascites management within 3 months after diagnosis) to delay disease progression; avoiding unnecessary invasive procedures or frequent hospitalizations for low-risk patients, improving quality of life while ensuring safety. This risk-based individualized strategy refines and improves the principle of “early intervention” rather than replacing it.

In scenarios with numerous data features and high collinearity, LASSO regression offers distinct advantages over other methods. First, LASSO automatically selects the most impactful features by shrinking coefficients of irrelevant variables to zero, thereby reducing model complexity and improving interpretability [23,47]. Moreover, it effectively mitigates multicollinearity, preventing overestimation of coefficients that plagues traditional regression approaches. Second, when integrated with the Cox proportional hazards (CPH) model (termed LASSO Cox), it can analyze time-dependent survival data without assuming a constant hazard ratio, a critical limitation of simpler models [48,49]. These advantages collectively make LASSO Cox a more robust and comprehensive tool for survival data analysis [23]. However, Lasso regression has its intrinsic limitations: its performance may decline with small sample sizes or extreme collinearity, warranting caution in such contexts.

This study had several limitations. First, it was a single-center study conducted in a single county, and given the regional and international variability in advanced schistosomiasis epidemiology and clinical manifestations, this inherently limited the generalizability of results-including the nomogram-to other healthcare settings and populations. Prospective multicenter studies are needed to validate the nomogram’s applicability globally. Second, a key limitation of LASSO regression is its tendency to arbitrarily exclude one of two highly correlated variables, a scenario where ridge regression might offer better stability. However, because ridge regression does not perform variable selection-critical for constructing a parsimonious nomogram where fewer variables are preferable-LASSO remained the more suitable choice for this analysis. Third, the relatively small sample size meant that stratifying patients based on restricted cubic spline (RCS) regression results might have obscured subtle but meaningful data trends. Finally, as a retrospective study, the nomogram was validated only internally, and the analysis focused exclusively on one clinical subtype of advanced schistosomiasis (ascites-predominant) in Dongzhi County. Future research should include prospective validation across diverse advanced schistosomiasis subtypes and larger populations to further refine and validate the nomogram’s predictive performance.

## Conclusion

Our study identified prognostic factors for advanced schistosomiasis and developed a nomogram using data from a retrospective cohort. Following variable selection via LASSO regression, four independent predictors-carbohydrate antigen 125, hyaluronic acid, ascites grade Ⅱ, and ascites grade Ⅲ-were incorporated into the nomogram. This tool has the potential to aid clinicians in accurately identifying advanced schistosomiasis patients at high mortality risk, thereby informing management decisions to reduce disease burden when integrated with appropriate treatment and follow-up strategies.

## Supporting information

S1 Fig Dongzhi County. The direct link to the base layer of the map: National Catalogue Service for Geographic Information (http://www.webmap.cn/commres.do?method=result100W). The link to the terms of use/ license information for the base layer image or shapefile: https://www.webmap.cn/main.do?method=otherService&clickFlag=service.
(TIF)

S1 Table Data of patients included in analysis. (XLSX)
