# Supplementary figures and images for: Prognostic determinants and mortality risk of advanced schistosomiasis revealed by Lasso-Cox regression integrative approach
Source: PLoS Negl Trop Dis. 2026 Jan 5;20(1):e0013846. doi: 10.1371/journal.pntd.0013846 (PMC12768372; doi:10.1371/journal.pntd.0013846)

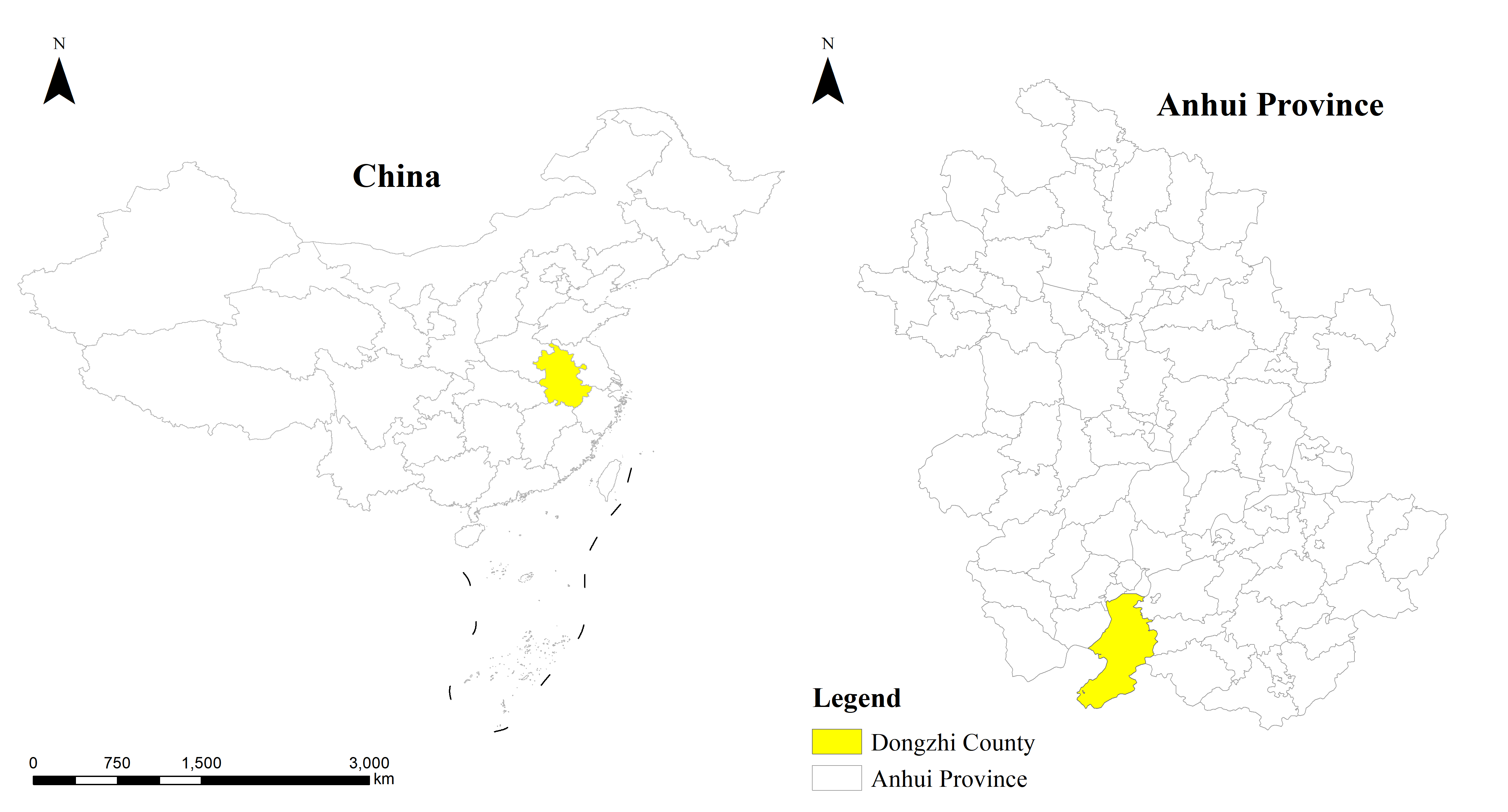

Supplement: S1 Fig — The direct link to the base layer of the map: National Catalogue Service for Geographic Information (http://www.webmap.cn/commres.do?method=result100W). The link to the terms of use/ license information for the base layer image or shapefile: https://www.webmap.cn/main.do?method=otherService&clickFlag=service. (TIF) [file pntd.0013846.s001.tif]
